# Real-World Safety of TwinRab, the World’s First Novel Cocktail of Rabies Monoclonal Antibodies, in a Clinical Setting

**DOI:** 10.7759/cureus.52163

**Published:** 2024-01-12

**Authors:** Asis Manna, Asis Kumar Kundu, Biswanath Sharma Sarkar, Baisakhi Maji, Trayambak Dutta, Manish Mahajan

**Affiliations:** 1 Microbiology, Infectious Diseases & Beliaghata General Hospital (IDBGH), Kolkata, IND; 2 Model Anti-Rabies Clinic, Infectious Diseases & Beliaghata General Hospital (IDBGH), Kolkata, IND; 3 General Medicine, Infectious Diseases & Beliaghata General Hospital (IDBGH), Kolkata, IND; 4 Community Medicine, Infectious Diseases & Beliaghata General Hospital (IDBGH), Kolkata, IND; 5 Medical Affairs, Zydus Lifesciences, Ahmedabad, IND

**Keywords:** adverse events, safety assessment, twinrabtm, post-exposure prophylaxis, rabies

## Abstract

Objectives: Every year, 18,000-20,000 people die from rabies in India, with children younger than the age of 15 accounting for 30%-60% of all cases. Wound cleaning, vaccination, and rabies immunoglobulin* (RIG)* administration are all part of treatment. TwinRab^TM^, a unique combination of two monoclonal antibodies (mAbs), docaravimab and miromavimab, effectively neutralizes rabies and rabies-like viruses. We conducted this study to evaluate the safety of the cocktail in patients infected with category-III animal bites according to WHO guidelines.

Methods: This open-label observational study was conducted in patients with WHO category-III animal bites by suspected rabid animals. All participants were screened, enrolled, and were administered the TwinRab^TM^ manufactured by Zydus Lifesciences Ltd. at the rate of 40 IU/kg by infiltration in and around the wound along with anti-rabies vaccine (ARVs). Participants were assessed at various intervals, and any adverse events (AEs) were documented and reported to the sponsor within 24 hours.

Result: The study enrolled 401 participants, 55.61% (n = 223) male, whose median age was 34 years. Adults made up 69.83% (n = 280) of the participants. The most exposed sites were the lower parts of the body (60.6%, n = 243); 99.75% (n = 400) of the population showed normal cardiovascular, gastrointestinal, and central nervous systems. After seven days of the last postexposure prophylaxis (PEP) dose, 9.98% of the total study population experienced 80 mild local solicited AEs and were assessed and treated.

Conclusion: The study concluded that TwinRab^TM^ when given in a 40 IU/kg dose with Essen or the updated Thai Red Cross Vaccine regimen, provides safe and effective rabies prophylaxis in WHO category III patients exposed to suspected rabied animal bites.

## Introduction

Human rabies is a serious public health issue and a zoonotic, viral disease that can be prevented through vaccination. It is estimated that canine rabies causes approximately 59,000 deaths worldwide each year [1]. Rabies is almost always fatal once the clinical symptoms appear. Domestic animals are responsible for rabies virus transmission to humans in up to 99% of cases. However, rabies can strike both domestic and wild animals. It is transmitted to humans and animals through bites or scratches, most commonly through saliva [2].

Rabies is found on all continents except Antarctica, with Asia and Africa accounting for more than 95% of human deaths. The WHO designates rabies as a neglected tropical disease (NTD) that primarily affects poor and vulnerable populations living in remote rural areas. Rural areas account for approximately 80% of human cases. Although there are effective human rabies vaccines and immunoglobulins, they are not widely available or accessible to those in need. Rabies deaths are rarely reported globally, and children aged five to 14 years are frequent victims [3]. According to the WHO, approximately 18,000 to 20,000 deaths occur in India each year. Children under the age of 15 account for 30%-60% of reported rabies cases and deaths in India, as bites in children frequently go unrecognized and unreported [4]. Cats (2%), jackals, mongooses, and other animals (1%) are responsible for approximately 97% of human rabies. The disease is primarily spread through the bite of a rabid animal. In a rabies-endemic country like India, where animal-to-animal transmission is prevalent, every animal bite is suspected of being a potentially rabid animal bite, and treatment should begin immediately after exposure [4]. Post-exposure prophylaxis (PEP) for bite victims has been established under the National Guidelines on Rabies Prophylaxis [4].

Single or multiple transdermal bites or scratches, contamination of mucous membranes or broken skin with saliva from animal licks, and exposures due to direct contact with rabid animals are all examples of WHO category III rabies exposure (severe exposure). The treatment, according to WHO guidelines, includes wound washing, immediate vaccination, and the administration of rabies immunoglobulin [1].

Post-exposure prophylaxis is the treatment given to a bite victim immediately following rabies exposure. It began with nerve tissue-derived vaccines, which were replaced in the 1960s by tissue culture-derived vaccines [5]. This prevents the entry of the virus into the central nervous system, which can result in death. Post-exposure prophylaxis entails thorough washing and local treatment of bite wounds or scratches as soon as possible following a suspected exposure and a course of potent and effective rabies vaccine that meets WHO standards, as well as rabies immunoglobulin (RIG) administration if indicated. Rabies immunoglobulins were developed to provide an immediate source of rabies-neutralizing antibodies, and their efficacy was demonstrated in Iran, the former Soviet Union, and China in addition to the rabies vaccine [6-9]. Rabies immunoglobulin products currently on the market are derived from immunized horses (equine rabies immunoglobulin (ERIG)) or humans (human rabies immunoglobulin (HRIG)). Despite the fact that both products pose a risk of bloodborne pathogens or adventitious agents and ERIG poses a risk of severe allergic reactions, ERIG is more commonly used due to its availability and lower cost.

In developing countries, cost is an important factor in the use of RIG in PEP [10]. A study in India found that only 21 of 783 (2.7%) patients with category III bites were prescribed HRIG, and only 10 could afford to obtain it [11]. Other studies from India and Thailand have also shown that only 2%-3% of patients with severe animal bites receive RIG [12]. As a result, it is not surprising that rabies mortality remains high. To address this critical issue, recombinant DNA technology was used to create a human monoclonal antibody (mAb) against the rabies virus glycoprotein (G).

The invention of the concept of mAb technology by Kohler and Milstein in 1975 revolutionized biomedicine [13]. The rationale for using mAbs for therapy is that they provide a more potent product with higher activity than polyclonal antibodies (pAbs). Furthermore, they do not appear to have inherent variability in terms of epitope and isotype and are homogeneous in nature, resulting in relatively low lot-to-lot variability. Significantly, the duration of action of mAbs can be predicted and is most likely related to the biological half-life. The high specificity of mAbs allows for the administration of a small amount of protein and volume, which eliminates the risk of several adverse events (AEs), including compartment syndrome [14].

Amongst mAbs, TwinRab^TM^ is a unique combination of two mAbs, docaravimab (62-71-3) and miromavimab (M777-16-3), that bind to two different epitopes on the G protein expressed on the surface of the rabies virus. Both mAbs bind to and neutralize rabies and rabies-like viruses, preventing them from infecting neighboring cells. Individual mAbs found in the TwinRab^TM^ cocktail mixture were found to neutralize various rabies and rabies-related viruses in vitro, including CVS 11, SAD B19, PV, Kelev, European fox, dog Turkey, dog Ethiopia, dog India, dog Mexico, wolf Sarajevo, bobcat-USA, EBLV 1, EBLV 2, East European fox, polar fox, dog Azerbaijan, and dog Nepal. The antibody cocktail was also shown to neutralize rabies virus strains isolated from dog, canine, human, and bovine sources in southern India [15]. If mAbs are being considered for rabies prevention, the WHO also recommends that at least two mAbs targeting different antigenic sites of the G protein be included in the use of antibody cocktails. As per the phase 3 study of Twinrab^TM^, it is found to be efficacious, safe, and non-inferior to HRIG. The FDA also recently published a document as a guide for the commercial development of such antibody cocktails [16].

In order to evaluate the safety of the combination of docaravimab and miromavimab in patients infected with category III animal bites in accordance with WHO guidelines, we conducted an open-label, single-armed controlled study in compliance with regulatory guidelines and recommendations for post-marketing surveillance studies for the approved indications.

## Materials and methods

Study design

Patients with suspected rabid animal bites (WHO category III) were the subjects of an open-label observational study. This single-arm study lacked a comparator group. Patients were screened and, if found eligible, enrolled after they provided written consent.

Participants

Patients who had been exposed to a suspected rabid animal under WHO category III had to be older than two years old and arrive for care after an exposure of no more than 72 hours or no more than 24 hours in the case of exposures to the face, neck, hand, or fingers. Acute or chronic, clinically significant pulmonary, endocrine, autoimmune, psychiatric, cardiovascular, hepatic, or renal functional abnormality, as determined by medical history and physical examination, any major congenital defects, history of thrombocytopenia, or known bleeding disorders were excluded from the study. Also excluded were patients who had previously received complete PEP or pre-exposure prophylaxis with the modern rabies vaccine. The study included 401 participants in total, and the findings showed that there was no justification for considering discontinuing it, and none of the participants had dropped out.

The study was carried out in accordance with Schedule Y, the Indian Council of Medical Research's (ICMR) Ethical Guidelines for Biomedical Research on Human Participants, the International Council for Harmonization's (ICH) Good Clinical Practice (ICHGCP) guidelines, the Central Drugs Standard Control Organization's (CDSCO) Good Clinical Practice (GCP) guidelines, and other applicable regulatory agencies.

All study participants provided informed consent, and parents or legal guardians did the same in the case of children. In accordance with regulatory requirements, the consent process was audio-visually recorded. According to current norms and regulations, the investigator had kept all legal records under lock and key, including source documents (SD), site master files (SMF), informed consent forms and documents (ICF/ICD), and other logs and forms.

Ethical committee approval

The study was registered in the Infectious Diseases & Beliaghata General Hospital (IDBGH), Kolkata, India on September 22, 2022 (registration number: IDBGH/Ethics/4150), and obtained approval from the Institutional Ethics Committee of the IDBGH. The study was registered in the Clinical Trials Registry- India (CTRI) (registration number: CTRI/2022/11/046994, registered on November 2, 2022).

Study drugs

The study drug is the combination of two murine anti-rabies mAbs called TwinRab^TM^, docaravimab (62-71-3), and miromavimab (M777-16-3). TwinRab^TM^ is a unique combination of two mAbs that bind two different epitopes on the G protein expressed on the surface of the rabies virus. The two mAbs bind to and neutralize both rabies and rabies-like viruses, preventing their infection of neighboring cells. They are manufactured by Zydus Lifesciences Ltd. (Ahmedabad, Gujarat, India).

Post-exposure prophylaxis administration and monitoring

**Figure 1:**
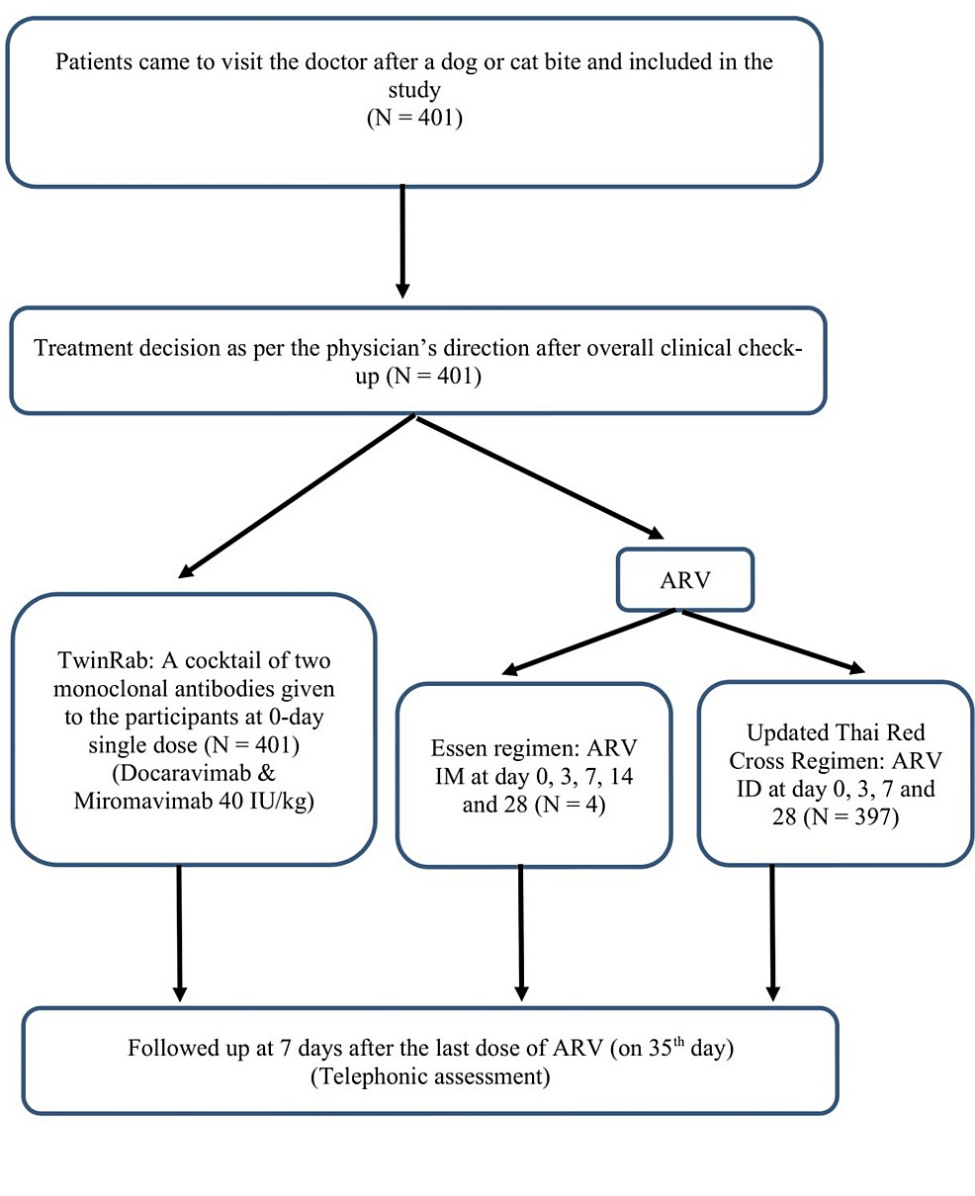
Study procedure for the world’s first cocktail of rabies monoclonal antibodies, TwinRabTM, in a clinical setting ARV: anti-rabies vaccine; IM: intramuscular; ID: intradermal

Each participant was administered the cocktail of monoclonal anti-rabies antibodies (docaravimab and miromavimab) on day 0 only with anti-rabies vaccines (ARV), Vaxirab N, which is a purified Chick Embryo Cell Vaccine (PCECV) manufactured by Zydus Lifesciences Ltd., as per the Essen regimen (intramuscular (IM)) on days 0, three, seven, 14, and 28 or the updated Thai Red Cross regimen (intradermal (ID)) on days 0, three, seven, and 28) as per the approved standard dosage regimen with ARV at the rate of 40 IU/kg body weight by infiltration around the bite wound/wounds. Rabies immunoglobulin was not used in the PEP provided to the patients. Any residual volume was delivered intramuscularly at a site separate from the vaccination injection site (Figure 1).

Participants were monitored for local injection site and systemic reactions for the first seven days. In addition, participants were to report any unsolicited AEs occurring during the 35-day study period. Clinical examinations were done in participants, and any AEs were evaluated at the scheduled time point (3+2, 7+2, 14+2, 28+2, and 35+7 days) as per the assessment table prepared by the investigator. Unscheduled visits were also allowed at any time during the study for the assessment and management of AEs and any concurrent clinical conditions. All AEs occurring during the course of the clinical study (i.e., from signing the informed consent onwards) were collected, documented, and reported by the investigator. Any serious or significant AEs, whether or not considered related to the investigational product, and whether or not the investigational product was administered, were reported immediately by telephone or fax to the sponsor, as per the respective regulatory requirements, within 24 hours.

Outcomes

The study's endpoint was day seven, after the last dosage of the PEP regimen. The occurrence of solicited adverse reactions (local AEs like pain, erythema, swelling, tenderness, and induration at the site of injection and systematic AEs like headache, malaise, arthralgia, myalgia, nausea, and vomiting), unsolicited nonserious and serious AEs, use of concomitant medications, and tolerability of a cocktail of monoclonal anti-rabies antibodies were all considered safety outcomes.

Data collection and statistical analysis

A clinical data management system (CDMS) was used to manage the study data, and data management was performed as per the standard operating procedures for CDM. All study-related data generated during the study were collected in manual case report forms (CRFs) and recorded in source documents. For statistical purposes, SAS® software version 9.4 (SAS Inc., Cary, NC) was used. The quantitative variables like age, height, weight, respiratory rate, blood pressure, and time since exposure were presented in median and range, and qualitative variables like gender, type of biting animal, type of wounds, body part involved, systemic examinations, vaccine regimen, presence and type of AEs that occurred during the study period, and tolerability of a cocktail of monoclonal anti-rabies antibodies were expressed as percentages and proportions.

## Results

A total of 401 participants were screened and enrolled in the study, and they all received the TwinRab^TM^ mAb cocktail. Among the 401 subjects, 55.61% (n=221) were male and 44.39% (n=178) were female. The participants' median age was 34 years, with a range of 18 to 50 years; median height was 154 cm, with a range of 149 to 161 cm; and median weight was 55 kg, with a range of 45 to 64 kg. Adults (18 to 65 years) contributed 69.83% (n=280) of the total participants, followed by children (five to 12 years) at 11.97% (n=48), adolescents at 7.73% (n=31), the geriatric age group (above 65 years) at 5.5% (n=22), and children (below five years) at 5% (n=20) (Tables 1, 2). As per our this study, the minimum pediatric age of subjects recruited was one year of age, and the maximum geriatric age was 90 years. We report 3800 IU as the maximum dose used in this study for Twinrab^TM ^without any AEs noted. We also report one subject, 0.24% (n=1), to be exposed to the saliva of a suspected rabid animal. The same was rinsed with Twinrab^TM^ for PEP.

**Table 1:** Baseline clinical and demographic details of participants (N=401) IQR: interquartile range

Variables	Median (IQR)
Age in years	34 (18.0 - 50.0)
Height in cm	154 (149 - 161)
Weight in kg	55 (45.0 - 64.0)
Gender	Frequency (percentage)
Male	223 (55.61%)
Female	178 (44.39%)
Vital signs	
Respiratory rate per minute (mean ± SD)	18 (17.0, 19.0)
Systolic blood pressure in mmHg (mean ± SD)	126 (120.0, 133.0)
Diastolic blood pressure in mmHg (mean ± SD)	83 (80.0, 92.0)

**Table 2:** Age-group-wise distribution of participants for biting animals, location of the bite on the body, and the vaccine regimen used (N = 401) *Essen PEP regimen (intramuscular) was administrated for four patients (age range: 18 to 65 years) due to their immunocompromised status

Age groups	<5 years (n=20, 4.99%)	≥5 and ≤ 12 years (n=48, 11.97%)	≥13 and ≤17 years (n=31, 7.73%)	≥18 and ≤65 years (n=280, 69.83%)	>65 years (22, 5.49%)
Biting animal					
Dog, n	9	28	20	175	17
Cat, n	11	20	11	101	5
Monkey, n	0	0	0	4	0
Bite location on the body					
Upper body, n	14	25	10	101	8
Lower body, n	6	23	21	179	14
Vaccine administration in the study subjects, n	20	48	31	280	22
Vaccine regimen					
Updated Thai Red Cross regimen, n	20	48	31	280*	22

At the end of the study, after seven days of the last PEP dose, we reported that overall, 9.98% of participants with AEs showed a total of 80 AEs, and all were local in nature. All these AEs were categorized as unassessable with no causal link to TwinRab^TM^ and mild to moderate in severity by the investigator and were resolved without any sequelae. All AEs resolved, and no severe or unsolicited AEs were reported. Of all these AEs, most were seen in the age group of 18 to 65 years (67.5%), followed by the age group of 13 to 17 years (12.5%), >65 years (10%), and 5% of AEs were reported in participants who were less than five years and five to 12 years of age (Table 3). We report no solicited systemic AEs in this study after the administration of TwinRab^TM ^such as fever. We also report no loss to follow-up for this study which signifies compliance to the treatment provided. No rabies breakthrough infection was reported throughout the course of follow-up in this study.

**Table 3:** Age-group-wise distribution of AEs AEs: adverse effects

Age groups	Local solicited AEs				
	Pain	Erythema	Swelling	Tenderness	Induration
<5 years	10%	-	5%	-	5%
≥5 and ≤12 years	2.08%	-	4.17%	2.08%	2.08%
≥13 and ≤17 years	9.68%	3.23%	9.68%	6.45%	6.45%
≥18 and ≤65 years	5.36%	1.07%	5.36%	3.93%	2.14%
>65 years	9.09%	4.55%	4.55%	13.64%	13.64%

## Discussion

Despite the availability of effective PEP regimens, human mortality rates due to rabies infection remain unacceptably high [17]. Failure of PEP, defined as a patient dying despite receiving the correct protocol on time, is extremely rare among the estimated 20 million people who receive PEP each year. The few reported PEP failures all occurred in developing countries, and nearly all involved one or more deviations from the WHO-recommended prophylaxis protocol [18]. Delays in seeking rabies prophylaxis, lack of or improper administration of RIG (e.g., failure to inject all bite sites), lack of or improper primary wound care, and/or poor-quality rabies vaccine are the most common deviations from the recommended protocol that result in death [19]. Even accelerated vaccination schedules are known to not eliminate the need for RIG after severe exposure [20].

Preclinical data have indicated that the TwinRab^TM^ is a promising candidate for use as an alternative to HRIG and ERIG in PEP. In this observational study, we present the first post-marketing clinical data for the TwinRab^TM^ mAb cocktail. The overall tolerability of TwinRab^TM^ was excellent and good in more than 90% of subjects, which was summarized as per the assessment table formulated by the investigators (80% in the <5-year age group; 83.33% in the five to 12-year age group; 90.32% in the 13 to 17 year age group; 90.71% in the 18 to 65 year age group; and 100% in the <65 year age group). In this study, no patient had PEP failure or had withdrawn from the study, which also shows the high tolerability of this cocktail mAb. Owing to the highest potency of TwinRab^TM^ (600 IU/ml), among all the available modalities of PEP, which include ERIG, HRIG, and single mAb, the volume required to be infiltrated is minimal, which contributes to its better local tolerability and fewer AEs. The lower volumes required also facilitate the infiltration of the complete required dose into the wound, which is critical for treatment success. [18]

In the US study, among local AEs, some injection site bruising was reported. However, other typical local reactions were not seen. Overall, fewer local reactions were observed than in a similar study investigating intramuscular administration of HRIG in healthy subjects [21]. In our study, an overall 10% of participants reported AEs, out of which all were solicited as local AEs with mild to moderate severity and resolved after treatment without any sequelae. In the RAB-M-A001 (USA) study, AEs like headache, dizziness, fatigue, and vomiting were seen in which healthy adults received intramuscular HRIG in combination with the rabies vaccine [21].

In an observational study involving German healthcare workers, the most frequent AEs reported after PEP with HRIG and the rabies vaccine were tiredness, malaise, headache, and dizziness [22]. In an experimental study involving 200 participants with WHO category III suspected rabies exposures who received Serum Institute of India (SII) rabies human monoclonal antibody (RMAb) or HRIG (1:1 ratio), no deaths, cases of rabies, or any other serious AEs were reported during the study period. They reported a total of 461 AEs, of which 83.7% were solicited events and 16.3% were unsolicited events [23]. Based on the persistence of symptoms during PEP, the investigators of similar studies concluded that strong headaches, tiredness, dizziness, and paraesthesia might be symptoms specific to rabies vaccination. One more study was performed in the Indian constituency with 57 patients. In RAB-M-A001, headache was more frequent after administration of CL184 alone than after administration in conjunction with the rabies vaccine, but in RAB-M-A002, only one subject in the CL184 40 IU/mL group reported headache [23].

In our study, we also had no reporting of any unsolicited AEs, which were seen at much lower incidence rates in RAB-M-A002 than in RAB-M-A001 with the administration of CL184 [23]. Other studies performed in Asia also observed lower rates of unsolicited AEs, which can most likely be attributed to cultural differences in the reporting of adverse effects [24-26].

The limitations of this study include the fact that it was carried out on a limited sample and in patients who had a possible exposure to rabies rather than patients who had a confirmed illness.

## Conclusions

Monoclonal antibodies have emerged as a promising replacement for RIGs due to their high effectiveness, elimination of animal use in the production process, standardized manufacturing, and reduced risk of adverse events. The introduction of TwinRab^TM^ represents a significant advancement in urgent PEP for category III suspected rabid animal bites. TwinRab^TM^ is the world's first innovative combination of two mAbs, docaravimab and miromavimab, which have been demonstrated to be well-tolerated for rabies PEP without significant adverse effects. The AEs were categorized as unassessable and showed no causal link to TwinRab^TM^. They were generally mild to moderate in severity, according to the investigator, and resolved without any lasting effects. TwinRab^TM ^received positive feedback from doctors and demonstrated good tolerability among patients.

This post-marketing surveillance study suggests TwinRab^TM^ to be a safe and effective alternative to human and equine-derived immunoglobulins. However, it is important to note that while this study is part of a large-scale real-world surveillance effort, larger clinical trials are needed to further confirm the safety of mAbs in rabies PEP.
